# Association analysis of CCL5, P2RX7, and SP110 gene polymorphisms with inactive pulmonary tuberculosis

**DOI:** 10.3389/fcimb.2026.1937385

**Published:** 2026-10-02

**Authors:** Fengting Chen, Haitao Wang, Junfeng Ren, Jifeng Cao, Chong Liu, Shanying Tong, Changdong Wu

**Affiliations:** 1 Xinjiang Emergency Center, People’s Hospital of Xinjiang Uygur Autonomous Region, Urumqi, Xinjiang, China; 2 Department of Respiratory Medicine, People’s Hospital of Xinjiang Uygur Autonomous Region, Xinjiang, China; 3 Department of Respiratory Medicine, The First Affiliated Hospital of Shihezi University, Shihezi, Xinjiang, China

**Keywords:** biomarkers, pulmonary tuberculosis, single nucleotide polymorphism (SNP), susceptibility genes, transcriptome

## Abstract

**Background:**

Host genetic background plays a crucial role in Mycobacterium tuberculosis infection and the development of pulmonary tuberculosis. This study aims to investigate the association between the polymorphisms of the *CCL5*, *P2RX7*, and *SP110* genes and the pathogenesis of inactive pulmonary tuberculosis.

**Methods:**

Identify hub genes based on GEO transcriptome data. A case-control study was conducted, enrolling 154 patients with inactive Pulmonary Tuberculosis (comprising 84 untreated and 70 treated cases) and 87 controls. Peripheral blood DNA was extracted, and six polymorphic loci within the *SP110*, *CCL5*, and *P2RX7* genes were genotyped using PCR and DNA sequencing. Univariate and multivariate logistic regression analyses were performed to evaluate genotypic and allelic distributions.

**Results:**

Two hub genes, *SP110* and *CCL5*, were identified from the transcriptome data. At the *SP110* rs9061 locus, the frequency of the A allele was lower in the untreated group compared to the control group, showing nominal significance (*P* = 0.015); however, this association did not remain significant after FDR correction (*P_FDR_* = 0.09). After adjusting for multiple factors, the A allele showed a nominally significant association with reduced IPTB risk (*P* = 0.042). Furthermore, although interaction (*P* = 0.682) and homogeneity tests (*P* = 0.584) were not statistically significant, limited subgroup sample sizes preclude claiming true homogeneity across subgroups. CMH stratified analysis revealed a nominally significant association (*P* = 0.034). Importantly, these adjusted results do not overcome the multiple-testing issue.

**Conclusions:**

The A allele at the *SP110* rs9061 locus may show a suggestive and exploratory association with decreased susceptibility to inactive pulmonary tuberculosis.

## 1 Introduction

TB remains a major global health challenge, with an estimated 8.3 million new cases in 2024 and a mortality rate roughly twice that of HIV/AIDS (Global Tuberculosis Report 2025, 2025). Notably, clinical outcomes following Mycobacterium tuberculosis (Mtb) infection exhibit profound heterogeneity. While an estimated one-quarter of the global population has a latent MTB infection, only 5% to 10% of infected individuals will develop active TB during their lifetime (Global Tuberculosis Report 2025, 2025). This relatively low progression rate and marked inter-individual variability strongly suggest that the host genetic background plays a pivotal regulatory role in TB susceptibility, disease progression, and ultimate prognosis (Tuberculosis, 2020).

Among the diverse clinical outcomes of MTB infection, inactive pulmonary tuberculosis (IPTB) represents a unique and crucial state. IPTB typically refers to individuals who have a history of TB or imaging features of lung lesions, but currently show no clinical or bacteriological evidence of active disease (Cheng et al., 2021). However, IPTB is not a completely benign endpoint; these individuals carry a significantly higher risk of TB relapse compared to the general population (Yu et al., 2022). Furthermore, the clinical trajectory of IPTB is deeply influenced by prior treatment history. Patients who achieved an inactive state through standardized anti-TB treatment may exhibit different immunological memory compared to those who spontaneously self-healed (Sossen et al., 2023). Therefore, understanding the genetic factors driving IPTB susceptibility, particularly under different treatment backgrounds, is critical for identifying high-risk populations and preventing relapse.

In this study, by integrating the Gene Expression Omnibus (GEO) database with machine learning algorithms, we identified *CCL5* and *SP110* as key genes associated with the disease. Recent reviews have highlighted that integrating machine learning with transcriptomic data has become a powerful and widely used strategy for host biomarker discovery in infectious diseases, including tuberculosis (Aalam et al., 2025; Aalam and Parveen, 2026). Furthermore, based on extensive previous literature, *P2RX7* was incorporated into our candidate gene set. Biologically, these genes play critical roles in the host’s immune response against MTB. The speckled 110 kDa (SP110) gene encodes the SP110 nuclear body protein, which mediates the host response to MTB at the nuclear level by regulating the apoptosis and necrosis of macrophages (Nakamura et al., 2024). C-C motif chemokine ligand 5 (CCL5) plays a crucial role in the immune response to Mycobacterium tuberculosis infection by recruiting T cells and monocytes to the site of infection (Novita et al., 2022). Purinergic receptor P2X, ligand-gated ion channel, 7 (*P2RX7*) acts as a “switch” in the clearance of intracellular bacteria by mediating apoptosis triggered by extracellular adenosine triphosphate (ATP) (Santiago-Carvalho et al., 2023). Given their essential functions in innate and adaptive immunity, single nucleotide polymorphisms (SNPs) in these genes may significantly alter host defense mechanisms and influence clinical stabilization into IPTB.

However, these findings, derived from bioinformatics analyses and prior research, warrant further experimental validation using clinical samples. The Xinjiang region is a high-burden area for TB in China. There is currently a paucity of systematic research focusing on this specific population, particularly studies that incorporate the critical variable of prior treatment history. To address this gap, we conducted a case-control study to investigate the association between *CCL5*, *P2RX7*, and *SP110* gene polymorphisms and the development of IPTB by comparing genetic susceptibility variations within the inactive PTB population.

## 2 Materials and methods

### 2.1 Bioinformatics analysis

#### 2.1.1 Dataset selection

Five TB-related microarray datasets (GSE19435, GSE19439, GSE19443, GSE28623, and GSE29536) were retrieved from the GEO database, normalized, and integrated to eliminate batch effects.

#### 2.1.2 DEG and enrichment analysis

Differentially expressed genes (DEGs) were identified using the R *limma* package (adjusted *P* < 0.05, |log2FC| > 1), followed by GO and KEGG functional enrichment via the DAVID database.

#### 2.1.3 Immune cell infiltration abundance

The CIBERSORT algorithm, using the LM22 reference matrix, evaluated the abundance of 22 immune cell subtypes.

#### 2.1.4 Machine learning

LASSO, SVM-RFE, and Random Forest algorithms were applied to the DEGs. The intersection of these three methods identified the final characteristic TB biomarkers.

#### 2.1.5 Construction and evaluation of the nomogram

It should be noted that feature selection for the SP110 and CCL5 variables was performed on the full dataset prior to model construction and evaluation. An exploratory TB risk nomogram was built using the rms package and its apparent performance was assessed via calibration curves, DCA, and ROC curves.

## 3 Clinical study

### 3.1 Study subjects

#### 3.1.1 Case group

A case-control study enrolled participants at the People’s Hospital of Xinjiang Uygur Autonomous Region. The case group comprised patients with IPTB, defined by characteristic CT manifestations, negative bacteriological tests, and an absence of active TB symptoms. Cases were further stratified by prior TB treatment history, whose ascertainment was performed through combined review of medical archives and patient-family interviews. Individuals with conditions such as HIV, malignancies, or immunosuppression were excluded. The control group included asymptomatic, smear-negative, long-term Xinjiang residents lacking radiological TB signs. The participant-selection flowchart and detailed imaging diagnostic criteria are shown in **Figures 1A, B**, respectively.

**Figure 1:**
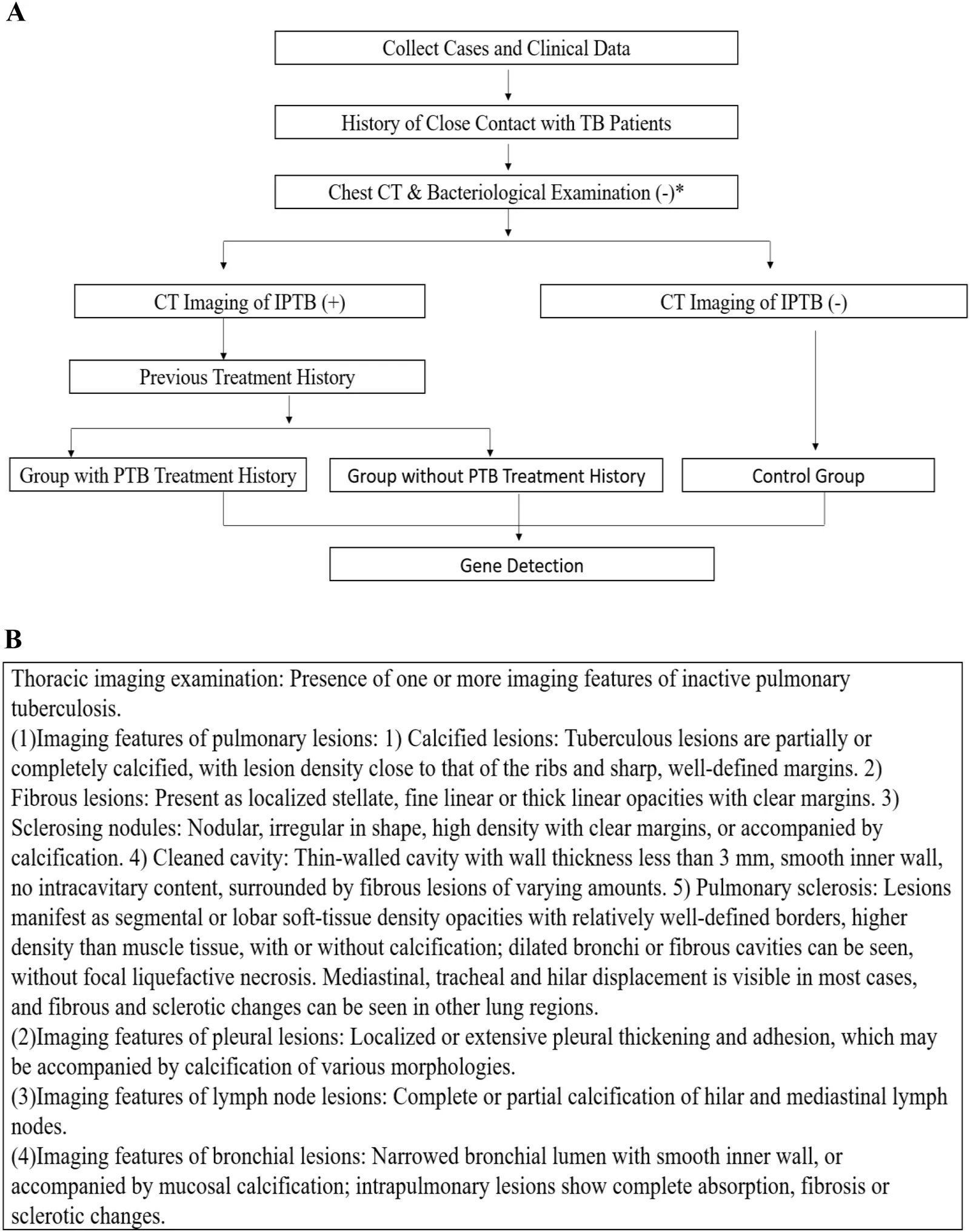
Flowchart of participant selection and the performance of the steps **(A)**; detailed radiological diagnostic criteria for IPTB **(B)**. IPTB, inactive pulmonary tuberculosis; *Full radiological descriptions of these criteria are presented in **(B)**.

### 3.2 Sample collection

Following the collection of comprehensive demographic and epidemiological data, 5 mL EDTA venous blood samples were drawn from all participants and cryopreserved at -80 °C for subsequent genomic DNA extraction.

### 3.3 Candidate TB susceptibility genes and their SNPs

Based on preliminary bioinformatics screening and secondary screening referencing the dbSNP and HapMap databases alongside existing literature, the candidate genes and key SNP loci associated with tuberculosis susceptibility were ultimately identified. Detailed information is provided in **Table 1**.

**Table 1:** Characteristics of candidate genes and SNP loci associated with TB susceptibility.

Gene	SNPs
SP110	rs9061 rs11556887 rs1135791
CCL5	rs2107538
P2RX7	rs1718119 rs3751143

### 3.4 Statistical methods

For each SNP, samples with failed genotyping were excluded on a per-marker basis; the number of missing calls per group is reported in the **Supplementary Tables**, and all tests were performed on the successfully genotyped subset. Continuous variables (mean ± SD) and categorical variables (counts/percentages) were analyzed using independent t-tests, and Chi-square or Fisher’s exact tests, respectively. Allele and genotype distribution differences were evaluated via Chi-square tests and univariate logistic regression. Loci with nominally significant associations were incorporated into a multivariate logistic regression model, adjusting for age, sex, ethnicity, and region as covariates. Nominal significance was set at a two-sided *P* < 0.05. To control false-positive rates, the Benjamini-Hochberg false discovery rate correction was applied separately within each pre-specified comparison stratum for both allele-level and genotype-level associations. Each hypothesis family contained the six target SNPs, yielding 10 separate correction families (six tests per family): five comparison strata (cases vs controls; untreated vs controls; treated vs untreated; treated vs controls; Uyghur subgroup vs Han subgroup), each assessed for both allele-level and genotype-level associations. Associations with an FDR-adjusted *P* < 0.05 were considered statistically significant. Analyses were performed using R (v4.5.2).

## 4 Results

### 4.1 Bioinformatics analysis results

#### 4.1.1 Identification of differentially expressed genes

To identify the DEGs between TB patients and healthy controls, transcriptome data from 87 control individuals and 117 TB patients were retrieved from the GEO database. PCA demonstrated a significant difference in the global gene expression profiles between the control and TB samples, indicating a trend of separation between the two groups (**Figure 2A**). According to the predefined screening criteria, a total of 80 up-regulated and 18 down-regulated DEGs were identified. To visually illustrate the distribution characteristics and expression patterns of these identified DEGs, a volcano plot (**Figure 2B**) and a hierarchical clustering heatmap (**Figure 2C**) were generated, respectively.

**Figure 2:**
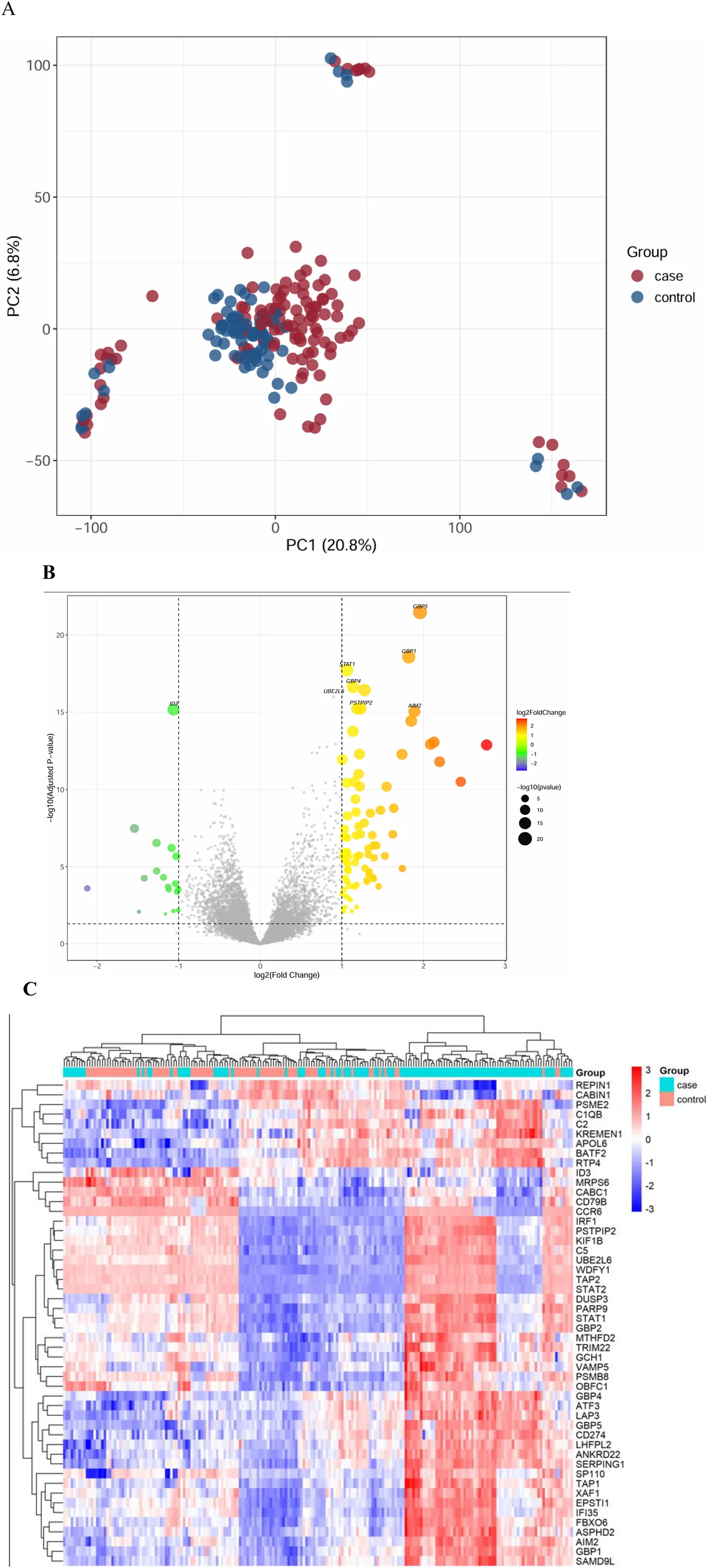
**(A)** PCA diagram showing the distribution of gene expression in case and control samples. **(B, C)** DEGs identified between control and case were compared with a volcano plot **(B)** and a heat map **(C)**. DEGs, differentially expressed genes.

DEGs were significantly enriched in GO BP terms related to immune and biotic responses (**Figure 3A**), CC terms involving cell surfaces and vesicles (**Figure 3B**), and MF terms concerning protein and immunoglobulin binding (**Figure 3C**). KEGG analysis revealed enrichment in infectious disease pathways, complement cascades, and NOD-like receptor signaling (**Figure 3D**).

**Figure 3:**
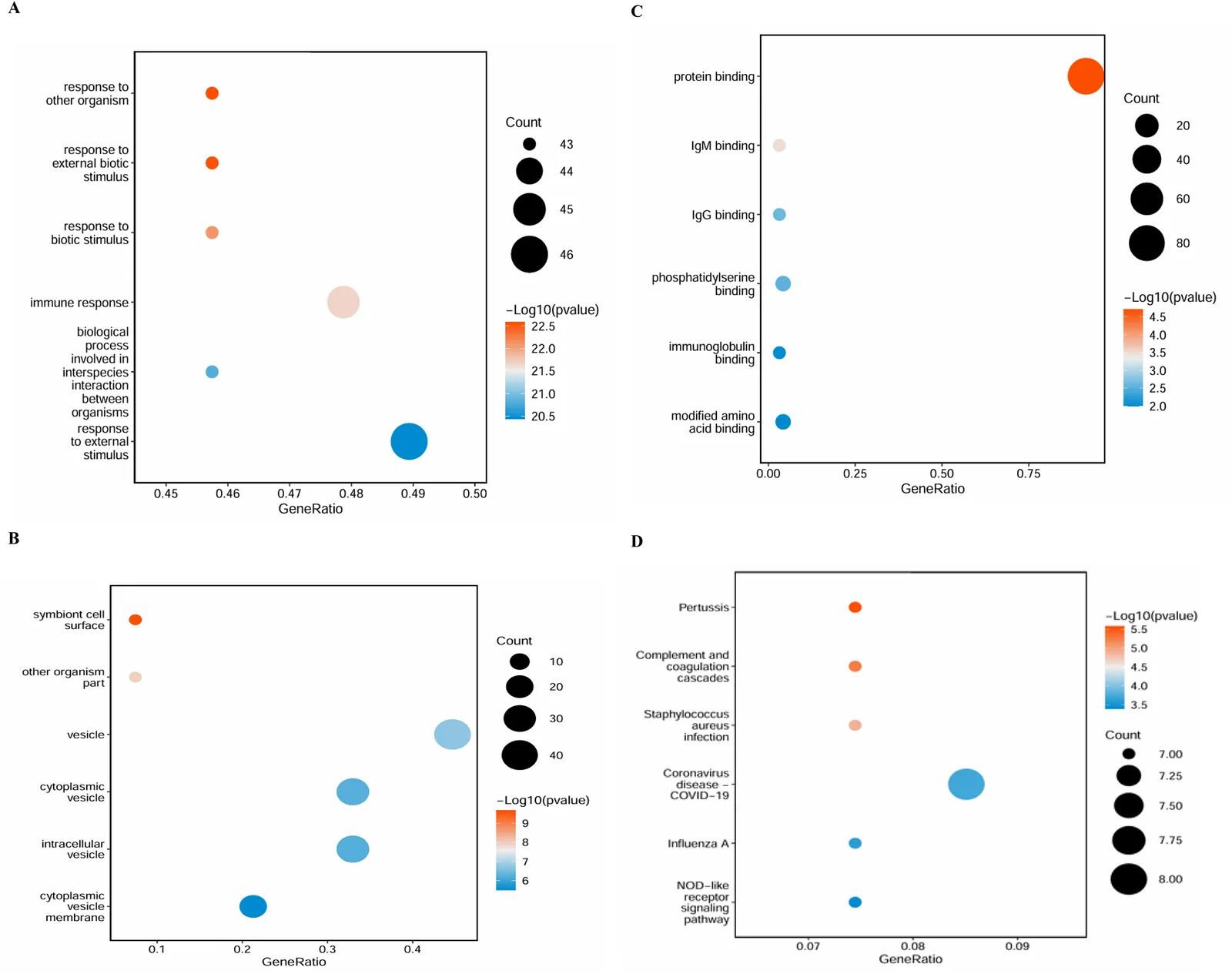
DEG functional enrichment analyses of GO BP **(A)**, GO CC **(B)**, GO MF **(C)**, and KEGG pathway terms **(D)**.

#### 4.1.2 Immune cell infiltration and correlation analysis

Compared to controls, the TB immune microenvironment shows significantly increased estimated proportions of pro-inflammatory innate cells (monocytes, neutrophils, M2 macrophages) and decreased estimated proportions of lymphocytes (CD8+ T, naive CD4+ T, naive B cells) (**Figure 4A**). Correlation analysis (**Figure 4B**) reveals complex co-occurrence networks, including positive M1/activated CD4+ T cell and negative neutrophil/CD8+ T cell associations. This inferred imbalance may reflect a distinct immunological profile associated with TB pathogenesis.

**Figure 4:**
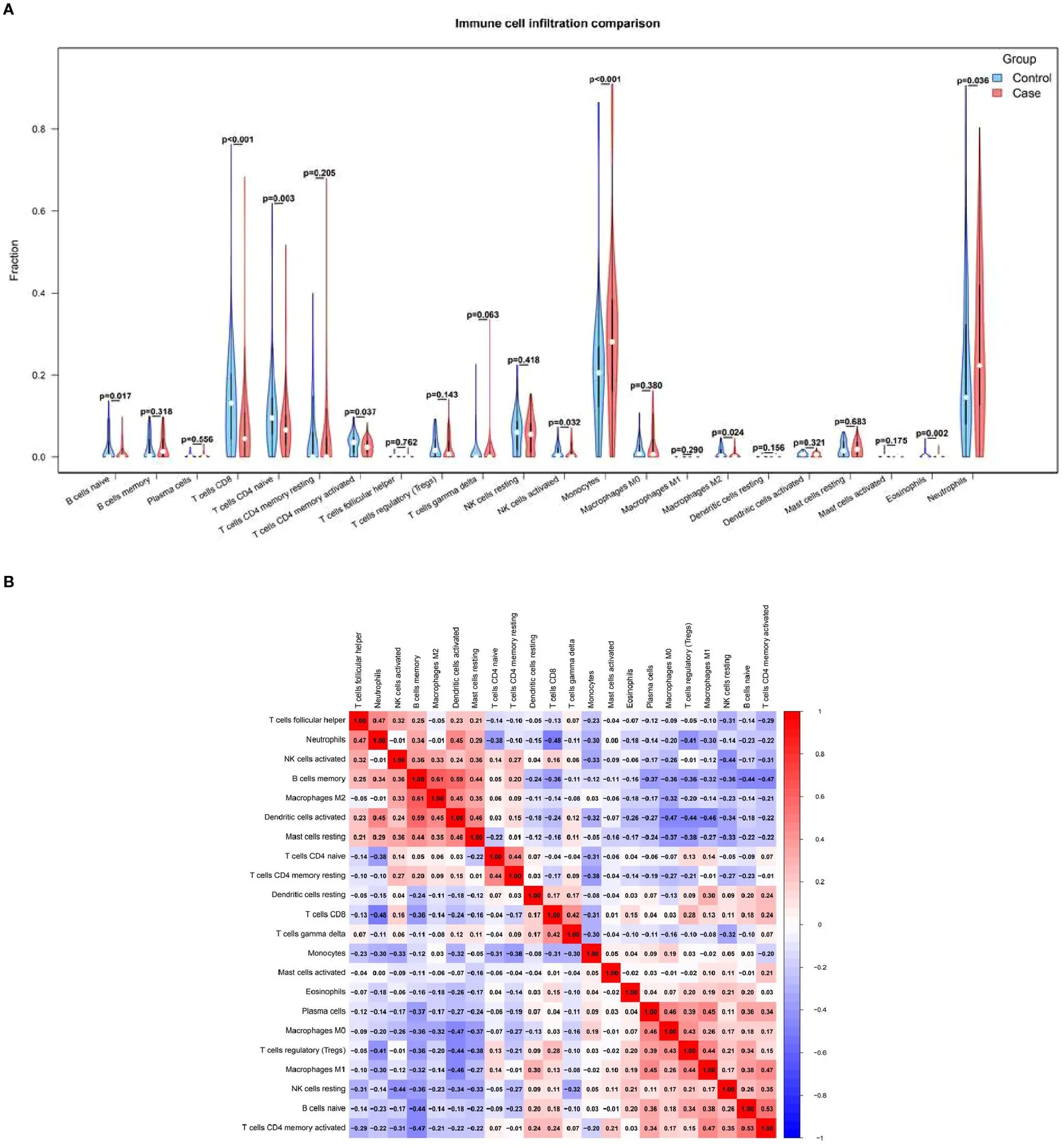
Immune cell enrichment in patients with tuberculosis **(A)**. Comparison of 22 immune cell subtypes between TB patients and controls **(B)**.

#### 4.1.3 Machine learning

To accurately identify key genes associated with TB, this study integrated three machine learning algorithms—LASSO regression, SVM-RFE, and Random Forest—for feature selection. By taking the intersection of the screening results from these three algorithms, and comprehensively considering feature importance and biological significance, *SP110* and *CCL5* were ultimately selected as the core variables for subsequent analysis and the construction of a predictive model (**Figures 5A–D**).

**Figure 5:**
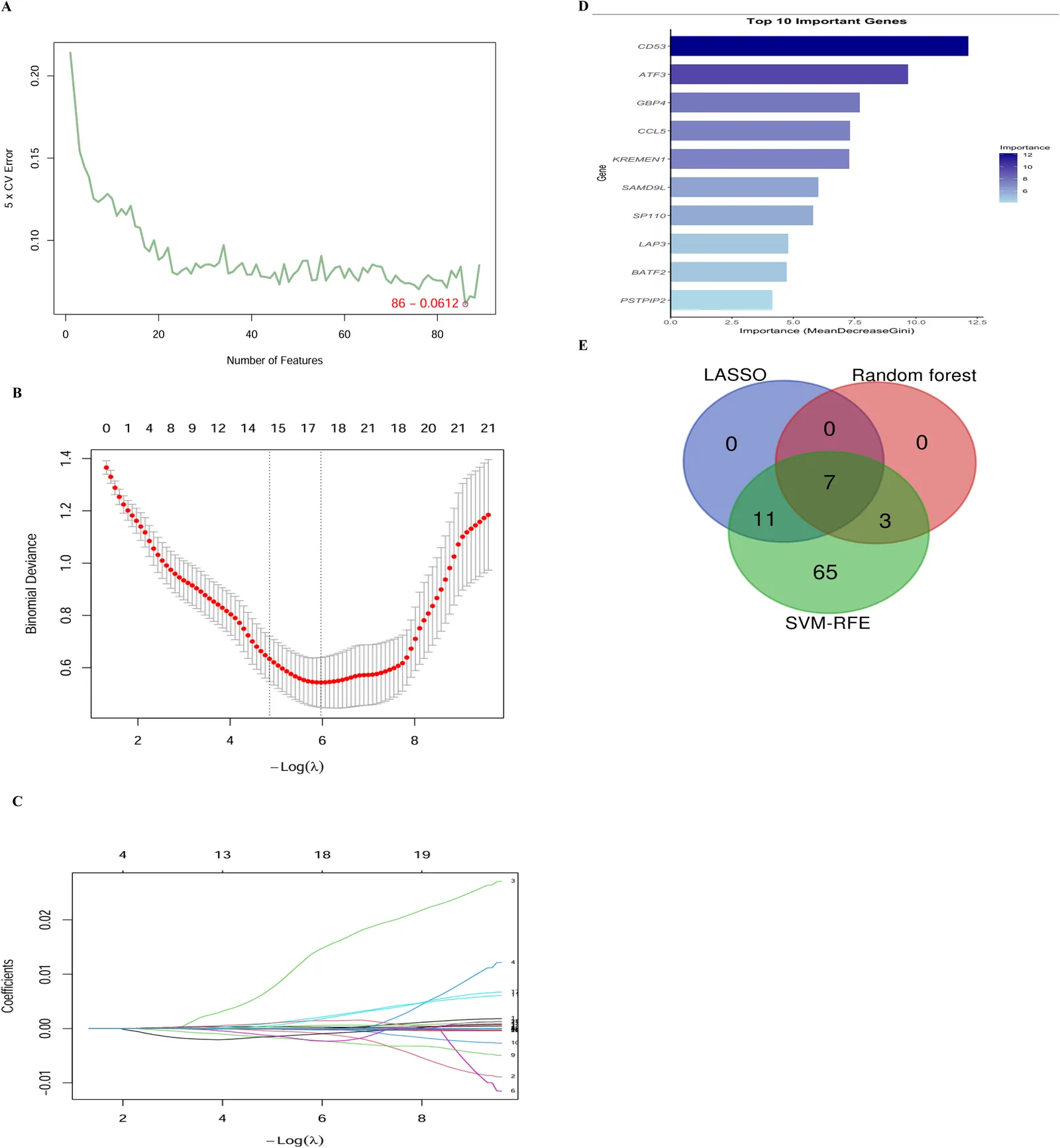
Gene feature selection related to tuberculosis based on machine learning. **(A–E)** Gene features associated with tuberculosis were constructed using SVM, LASSO regression, and RF methods.

#### 4.1.4 Construction and evaluation of the nomogram

Based on SP110 and CCL5, we constructed a TB risk nomogram (**Figure 6A**). Higher SP110 and lower CCL5 levels were associated with elevated TB risk. The model showed moderate apparent performance within this analyzed dataset (AUC = 0.747, **Figure 6B**), acceptable calibration (**Figure 6C**), and observed net benefit within this specific cohort (**Figure 6D**). Notably, no independent external validation was performed; therefore, the reported metrics solely represent apparent performance within the analyzed dataset rather than independently validated predictive performance. In summary, these results indicate the potential exploratory value of SP110 and CCL5 for TB-risk prediction, yet findings must be interpreted with considerable caution.

**Figure 6:**
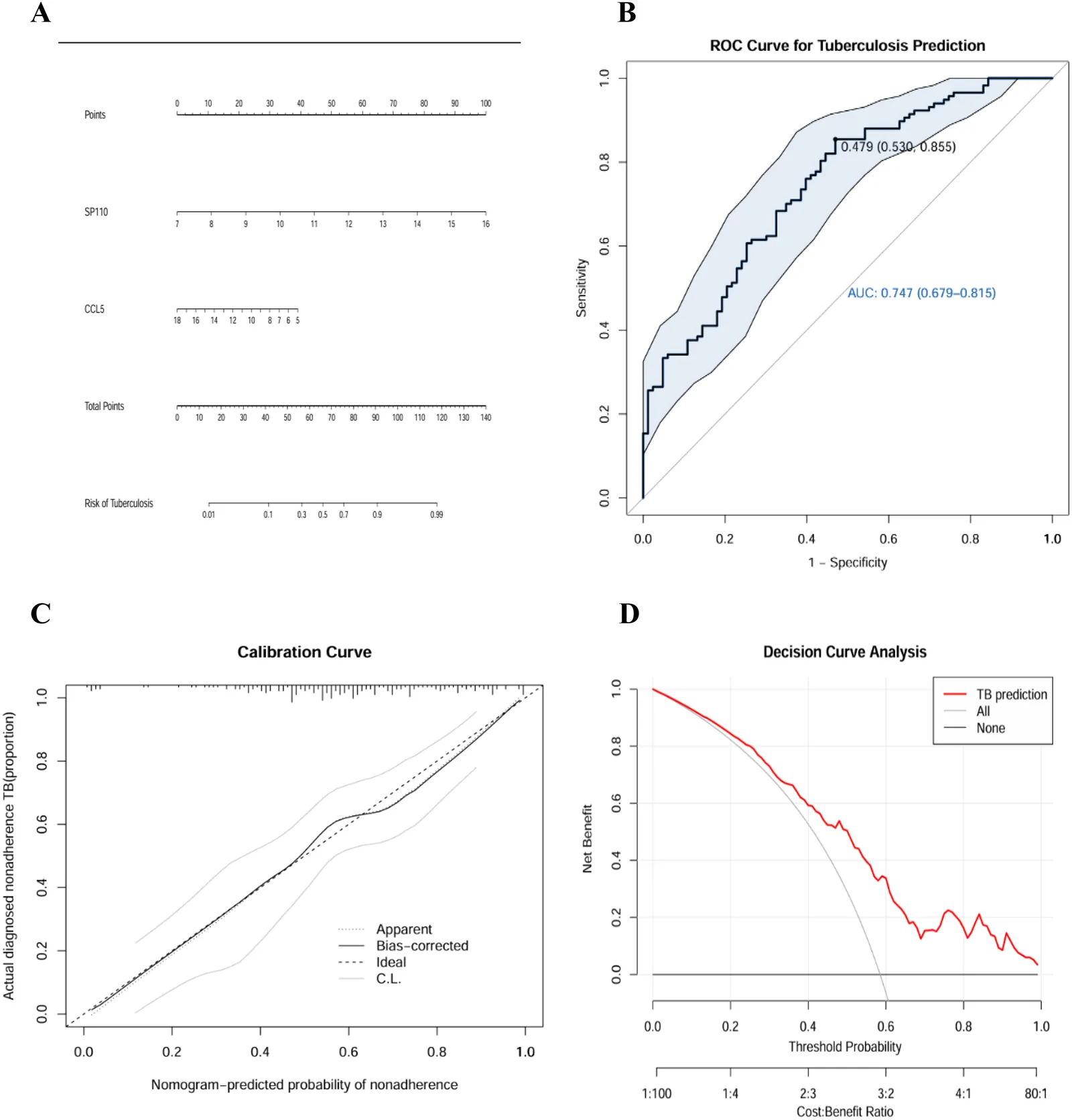
**(A)** A nomogram based on two genes for predicting tuberculosis risk. **(B)** The Receiver Operating Characteristic (ROC) curve of the model for diagnosing tuberculosis. **(C)** A calibration curve evaluating the predictive consistency of the nomogram. **(D)** Decision Curve Analysis (DCA) assessing the observed net benefit of the nomogram in this cohort.

### 4.2 Clinical research results

#### 4.2.1 General characteristics of the study subjects

We enrolled 241 subjects: 87 controls and 154 TB patients (84 untreated, 70 treated). Gender distribution was balanced (*P* = 0.966). However, age (*P* < 0.001) and regional demographics (*P* = 0.024) differed. The untreated TB cohort had the highest mean age and the largest proportion of Southern residents. Conversely, controls had the lowest mean age and predominantly originated from the Northern region (**Table 2**).

**Table 2:** General characteristics of the study subjects.

Variable	Overall N = 241^1^	Control N = 87^1^	Untreated N = 84^1^	Treated N = 70^1^	*P*
Age (years)	71.5 ± 10.1	68.1 ± 10.4	74.8 ± 8.9	71.9 ± 9.9	<0.001
Gender N (%)					0.966
Female	112 (46)	40 (46)	40 (48)	32 (46)	
Male	129 (54)	47 (54)	44 (52)	38 (54)	
Race N (%)					0.083
Han	78 (32)	24 (28)	24 (29)	30 (43)	
Uyghur	163 (68)	63 (72)	60 (71)	40 (57)	
Region N (%)					0.024
Northern	117 (49)	52 (60)	33 (39)	32 (46)	
Southern	124 (51)	35 (40)	51 (61)	38 (54)	

^1^ Mean ± SD; n (%) ^2^One-way analysis of means; Pearson’s Chi-squared test.

Northern, Northern Xinjiang; Southern, Southern Xinjiang.

#### 4.2.2 Genomic DNA extraction and PCR amplification

Whole blood genomic DNA extraction yielded high-quality, high-purity samples (OD260/280: 1.8–2.0) with clear, degradation-free bands confirmed via electrophoresis, fully meeting subsequent experimental requirements. Furthermore, PCR successfully amplified the targeted loci across the *SP110* (rs9061, rs11556887, rs1135791), *CCL5* (rs2107538), and *P2RX7* (rs1718119, rs3751143) genes. The resulting amplicons ranged from 370 bp to 696 bp, demonstrating specific and accurate amplification against a 100–2000 bp reference size marker (**Supplementary Figure 1**).

#### 4.2.3 Sanger sequencing genotyping results

Bidirectional sequencing of purified amplicons revealed three genotypes per locus. Chromatograms confirmed A/G alleles for *SP110* (rs9061, rs11556887, rs1135791) and *CCL5* (rs2107538); C/T for *P2RX7* rs1718119; and G/T for *P2RX7* rs3751143, displaying distinct homozygous and heterozygous peaks (**Supplementary Figure 2**).

#### 4.2.4 Hardy-Weinberg equilibrium test analysis results

The genotype frequencies of the evaluated SNPs in the SP110 (rs9061, rs11556887, rs1135791), CCL5 (rs2107538), and P2RX7 (rs1718119, rs3751143) genes conformed to Hardy-Weinberg equilibrium in both the case and control groups (*P* > 0.05), indicating no obvious deviation from HWE in our study populations. (**Supplementary Table 1**).

#### 4.2.5 Association between gene polymorphisms and IPTB susceptibility

##### 4.2.5.1 Association study of *SP110* gene polymorphisms and IPTB susceptibility

In this study, the genotype frequency distributions of the rs9061, rs11556887, and rs1135791 loci were statistically compared between the IPTB case group and the control group. The results showed that there was no significant association between *SP110* gene polymorphisms and susceptibility to IPTB in Xinjiang (*P* > 0.05).

To further explore the cross-sectional associations between gene polymorphisms and subgroups defined by treatment history, the study compared the differences in SP110 genotype and allele frequencies between IPTB patients with and without a history of treatment. (**Tables 3**–**5**; **Supplementary Table 1**).

**Table 3:** Untreated and control group alleles association study.

SNP	Alleles	Untreated (2N = 168) n (%)	Control (2N = 174) n (%)	χ^2^	*P*	*OR*	*95% CI*	*P_FDR_*
rs9061	A	17 (10.5)	35 (20.1)	5.937	0.015	0.466	[0.247~0.869]	0.090
	G	145 (89.5)	139 (79.9)			1	Ref	
	Missing	6	0					
rs11556887	A	11 (6.5)	17 (9.9)	1.252	0.263	0.639	[0.286~1.407]	0.526
	G	157 (93.5)	155 (90.1)			1	Ref	
	Missing	0	2					
rs1135791	A	126 (75.0)	132 (75.9)	0.034	0.853	0.955	[0.588~1.561]	0.891
	G	42 (25.0)	42 (24.1)			1	Ref	
	Missing	0	0					
rs2107538	A	44 (26.2)	51 (29.7)	0.506	0.477	0.842	[0.522~1.353]	0.715
	G	124 (73.8)	121 (70.3)			1	Ref	
	Missing	0	2					
rs1718119	C	128 (77.1)	138 (82.1)	1.305	0.253	0.732	[0.426~1.251]	0.526
	T	38 (22.9)	30 (17.9)			1	Ref	
	Missing	2	6					
rs3751143	G	33 (19.6)	34 (20.2)	0.019	0.891	0.963	[0.565~1.645]	0.891
	T	135 (80.4)	134 (79.8)			1	Ref	
	Missing	0	6					

**Table 4:** Untreated and treated group alleles association study.

SNP	Alleles	Treated (2N = 140) n (%)	Untreated (2N = 168) n (%)	*χ^2^*	*P*	*OR*	*95% CI*	*P_FDR_*
rs9061	A	27 (20.1)	17 (10.5)	5.403	0.020	2.152	[1.117~4.148]	0.120
	G	107 (79.9)	145 (89.5)			1	Ref	
	Missing	6	6					
rs11556887	A	16 (11.6)	11 (6.5)	2.398	0.121	1.872	[0.836~4.179]	0.242
	G	122 (88.4)	157 (93.5)			1	Ref	
	Missing	2	0					
rs1135791	A	97 (69.3)	126 (75.0)	1.248	0.264	0.752	[0.454~1.240]	0.317
	G	43 (30.7)	42 (25.0)			1	Ref	
	Missing	0	0					
rs2107538	A	37 (26.4)	44 (26.2)	0.002	0.962	1.012	[0.608~1.680]	0.962
	G	103 (73.6)	124 (73.8)			1	Ref	
	Missing	0	0					
rs1718119	C	118 (85.5)	128 (77.1)	3.443	0.064	1.752	[0.964~3.180]	0.192
	T	20 (14.5)	38 (22.9)			1	Ref	
	Missing	2	2					
rs3751143	G	36 (26.1)	33 (19.6)	1.802	0.180	1.444	[0.844~2.472]	0.270
	T	102 (73.9)	135 (80.4)			1	Ref	
	Missing	2	0					

**Table 5:** Treated and control group alleles association study.

SNP	Alleles	Treated (2N = 140) n (%)	Control (2N = 174) n (%)	*χ2*	*P*	*OR*	*95% CI*	*P_FDR_*
rs9061	A	27 (20.1)	35 (20.1)	0.000	0.994	1.002	[0.571~1.757]	0.994
	G	107 (79.9)	139 (79.9)			1	Ref	
	Missing	6	0					
rs11556887	A	16 (11.6)	17 (9.9)	0.236	0.627	1.196	[0.580~2.463]	0.752
	G	122 (88.4)	155 (90.1)			1	Ref	
	Missing	2	2					
rs1135791	A	97 (69.3)	132 (75.9)	1.699	0.192	0.718	[0.436~1.183]	0.675
	G	43 (30.7)	42 (24.1)			1	Ref	
	Missing	0	0					
rs2107538	A	37 (26.4)	51 (29.7)	0.396	0.529	0.852	[0.518~1.403]	0.752
	G	103 (73.6)	121 (70.3)			1	Ref	
	Missing	0	2					
rs1718119	C	118 (85.5)	138 (82.1)	0.627	0.428	1.283	[0.692~2.377]	0.752
	T	20 (14.5)	30 (17.9)			1	Ref	
	Missing	2	6					
rs3751143	G	36 (26.1)	34 (20.2)	1.469	0.225	1.391	[0.815~2.375]	0.675
	T	102 (73.9)	134 (79.8)			1	Ref	
	Missing	2	6					

Analysis of the *SP110* rs9061 locus showed that the A allele frequency was lower in the untreated group compared to controls (10.5% vs. 20.1%, nominal P = 0.015; *P_FDR_* = 0.09). Additionally, treated patients showed a higher A allele frequency than untreated patients (20.1% vs. 10.5%, nominal P = 0.020; *P_FDR_* = 0.12). No significant differences were observed between the treated and control groups. Loci rs11556887 and rs1135791 exhibited no significant differences between patient subgroups.

##### 4.2.5.2 Association study of *CCL5* and *P2RX7* gene polymorphisms

*CCL5* (rs2107538): No significant differences were detected in the distribution of genotype or allele frequencies between IPTB cases and controls. Stratified analysis also revealed no statistical association across the untreated and treated subgroups (**Tables 3**, **4**; **Supplementary Table 1**).

*P2RX7* (rs1718119 and rs3751143): General analysis showed no significant association for either locus with the total study population.

##### 4.2.5.3 Association study of SNPs between populations

We investigated the genetic polymorphisms of six candidate SNPs in Uyghur and Han populations. Genotype distribution analysis revealed a difference at the rs1718119 locus between the two ethnic groups (*P* < 0.01), which remained significant after false discovery rate correction (*P_FDR_* = 0.006). Other loci showed no significant genotypic differences after multiple testing correction (**Table 6**).

**Table 6:** Uyghur and Han group genotypes association study.

SNP	Alleles	Group	Genotype n (%)			Missing (n)	*P*	*P_FDR_*
	(1/2)		1/1	1/2	2/2			
rs9061	G/A	Uyghur (N = 163)	121 (75.6)	34 (21.2)	5 (3.1)	3	0.025	0.074
		Han (N = 78)	44 (58.7)	27 (36.0)	4 (5.3)	3		
rs11556887	G/A	Uyghur (N = 163)	135 (83.9)	24 (14.9)	2 (1.2)	2	0.809	0.809
		Han (N = 78)	63 (80.8)	14 (17.9)	1 (1.3)	0		
rs1135791	A/G	Uyghur (N = 163)	82 (50.3)	65 (39.9)	16 (9.8)	0	0.063	0.125
		Han (N = 78)	51 (65.4)	24 (30.8)	3 (3.8)	0		
rs2107538	G/A	Uyghur (N = 163)	89 (54.9)	65 (40.1)	8 (4.9)	1	0.134	0.202
		Han (N = 78)	36 (46.2)	33 (42.3)	9 (11.5)	0		
rs1718119	C/T	Uyghur (N = 163)	95 (59.0)	57 (35.4)	9 (5.6)	2	<0.001	0.003
		Han (N = 78)	63 (84.0)	11 (14.7)	1 (1.3)	3		
rs3751143	T/G	Uyghur (N = 163)	99 (61.9)	55 (34.4)	6 (3.8)	3	0.656	0.787
		Han (N = 78)	46 (59.7)	26 (33.8)	5 (6.5)	1		

In the allelic frequency analysis, three SNPs exhibited statistical difference. The T allele of rs1718119 was enriched in the Uyghur population compared to the Han group (23.3% vs. 8.7%, *P_FDR_* = 0.006). Similarly, the G allele of rs1135791 presented a higher frequency in Uyghurs (29.8% vs. 19.2%, *P_FDR_* = 0.028). Conversely, the A allele of rs9061 was less prevalent in the Uyghur cohort (13.8% vs. 23.3%, *P_FDR_* = 0.028). The remaining polymorphisms (rs11556887, rs2107538, and rs3751143) displayed no statistical differences (**Table 7**).

**Table 7:** Uyghur and Han group alleles association study.

SNP	Alleles	Uyghur (2N = 326) n (%)	Han (2N = 156) n (%)	*χ2*	*P*	*OR*	*95% CI*	*P_FDR_*
rs9061	A	44 (13.8)	35 (23.3)	6.708	0.010	0.524	[0.320~0.859]	0.028
	G	276 (86.2)	115 (76.7)			1	Ref	
	Missing	6	6					
rs11556887	A	28 (8.7)	16 (10.3)	0.306	0.580	0.833	[0.437~1.591]	0.580
	G	294 (91.3)	140 (89.7)			1	Ref	
	Missing	4	0					
rs1135791	G	97 (29.8)	30 (19.2)	6.022	0.014	1.779	[1.119~2.828]	0.028
	A	229 (70.2)	126 (80.8)			1	Ref	
	Missing	0	0					
rs2107538	A	81 (25.0)	51 (32.7)	3.125	0.077	0.686	[0.452~1.043]	0.115
	G	243 (75.0)	105 (67.3)			1	Ref	
	Missing	2	0					
rs1718119	T	75 (23.3)	13 (8.7)	14.431	<0.001	3.200	[1.713~5.977]	0.006
	C	247 (76.7)	137 (91.3)			1	Ref	
	Missing	4	6					
rs3751143	G	67 (20.9)	36 (23.4)	0.364	0.546	0.868	[0.548~1.375]	0.580
	T	253 (79.1)	118 (76.6)			1	Ref	
	Missing	6	2					

#### 4.2.6 Multivariate logistic regression analysis

Candidate loci were preselected based on a univariate screening threshold of *P* < 0.05 and then incorporated into multivariate models that adjusted for age, sex, race, and region.

##### 4.2.6.1 Multivariable-adjusted and interaction analyses of genetic associations (untreated versus control)

Multivariable logistic regression showed that after controlling for ethnicity, age, region and sex, an increasing dosage of the rs9061 A allele showed nominally associated with reduced risk of IPTB (*P* = 0.042). Notably, this association lost statistical significance following FDR correction. In addition, this model identified marked associations between disease status and age (*P* < 0.001) as well as region (*P* = 0.005).

To further explore potential synergistic or antagonistic relationships between genetic factors and ethnicity, an interaction term of rs9061 and ethnicity was incorporated into the logistic regression model. While no significant interaction was detected, due to the limited subgroup sample sizes, this absence of significance should not be strictly interpreted as demonstrating that the genetic effect is completely homogeneous across the Han and Uyghur subgroups.

Considering the multi-ethnic settlement characteristics of Xinjiang and the inclusion of two ethnic groups in our study, population genetic heterogeneity may introduce confounding bias in genetic association analysis. To further assess the consistency of the association across ethnic strata, the Breslow–Day test was performed, and no significant heterogeneity was observed (χ2 = 0.300, df=1, *P* = 0.584), which supported the application of CMH stratified analysis. After stratification by ethnicity, the pooled OR of rs9061 was 0.442 (95%CI: 0.219–0.892), with a nominally significant association (*P* = 0.034) (**Table 8**; **Figure 7**). Importantly, consistent with our univariate findings, these nominally significant multivariable and CMH results should not be interpreted as overcoming the multiple-testing issue.

**Table 8:** Multiple-model association analyses of rs9061 (untreated vs control).

Model	rs9061 A allele OR (95% CI)	P value
Crude model	0.466 [0.247–0.869]	0.015
Model I	0.442 [0.219–0.892]	0.034
Model II	0.500 [0.250–0.964]	0.042
Model III	0.545 [0.250–1.189]	0.127
rs9061 × ethnicity (interaction term)	0.735	0.682

Crude model, unadjusted allelic association. Model I, Cochran–Mantel–Haenszel test stratified by ethnicity. Model II, multivariable additive logistic regression adjusted for ethnicity, age, sex and region. Model III, additive logistic regression with additional rs9061-by-ethnicity interaction term. OR, odds ratio; 95%CI, 95% confidence interval. G allele was set as the reference in crude model.

**Figure 7:**
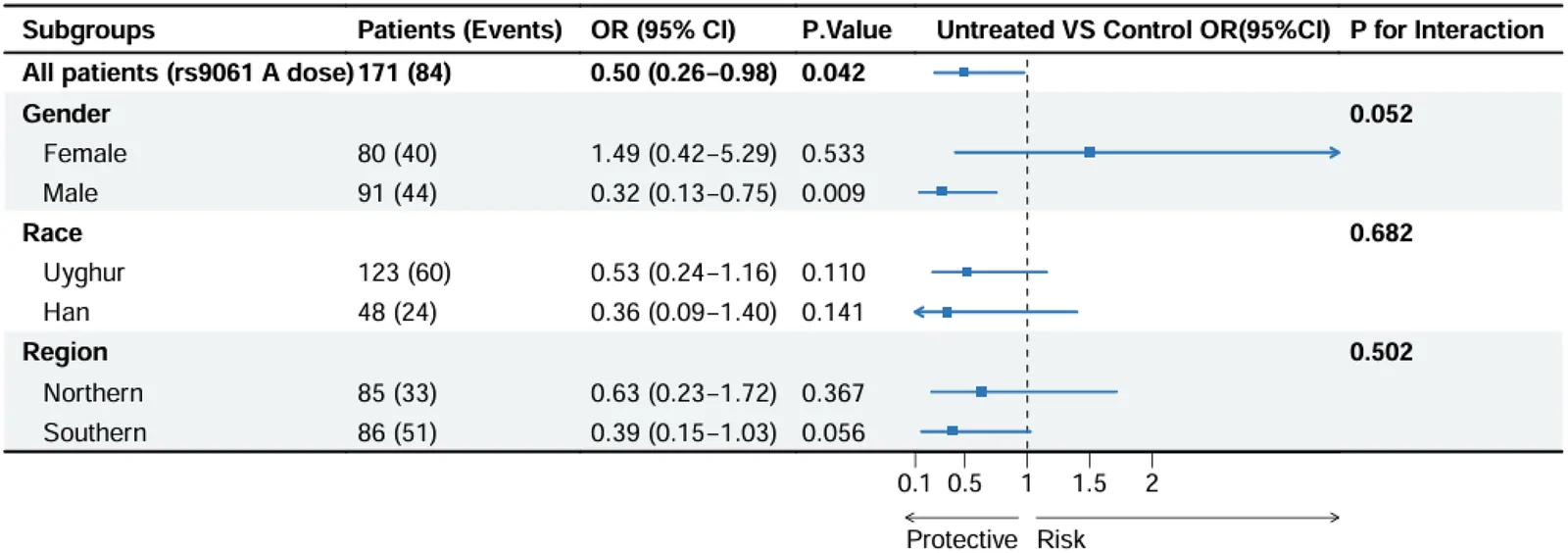
Subgroup analysis of rs9061 A allele dose and outcome.

##### 4.2.6.2 Multivariable-adjusted and interaction analyses of genetic associations (untreated versus treated)

Multivariable logistic regression showed that age had an independent association with cross-sectional treatment-history subgroup membership (*P* = 0.0149). After adjusting for confounders, the association related to the dosage of the rs9061 A allele was attenuated. The association observed in univariate analysis was no longer statistically significant and only showed a weak marginal trend (*P* = 0.0836). The CMH test stratified by ethnicity also did not identify a significant association (*P* = 0.1362). These observations suggest that the nominally significant association identified in univariate analysis may be influenced by baseline factors such as age; the genetic association was attenuated after accounting for baseline imbalance between groups. We further assessed whether ethnicity modified this genetic association. The Breslow–Day test for homogeneity (*P* = 0.102) and the interaction term test in multivariable regression (*P* = 0.1164) produced consistent results, with all *P* > 0.05. However, given the limited subgroup sample sizes, this lack of statistical significance in the interaction and homogeneity tests should not be strictly interpreted as demonstrating that the genetic effect is completely homogeneous across ethnic subgroups. (**Supplementary Table 1**).

Forest plot showing OR and 95% CI for the association between rs9061 A allele and IPTB risk in overall population and subgroups stratified by gender, race and region. The horizontal lines represent 95% CIs, and the squares denote OR values. P for interaction indicates the significance of interaction between rs9061 A allele dose and each subgroup variable. OR < 1 represents a protective effect against non-active tuberculosis. Northern = Northern Xinjiang; Southern = Southern Xinjiang.

## 5 Discussion

The pathogenesis of TB exhibits significant individual genetic heterogeneity; however, studies investigating genetic polymorphisms among IPTB populations in Xinjiang, particularly concerning diverse treatment histories, remain scarce. This study aims to investigate the association between the polymorphisms of the *CCL5*, *P2RX7*, and *SP110* genes and the susceptibility to IPTB in the Xinjiang region.

Through comparative transcriptomic analysis, this study identified 98 DEGs, comprising 80 upregulated and 18 downregulated genes. These DEGs are predominantly enriched in antigen presentation and inflammatory signaling pathways. In addition, immune infiltration analysis based on CIBERSORT-estimated cell proportions indicated a potential systemic immune imbalance in patients: the estimated relative abundance of pro-inflammatory cells such as neutrophils was increased, and macrophages exhibited a potential shift toward the immunosuppressive M2 phenotype. In contrast, the estimated proportions of cytotoxic adaptive lymphocytes (CD8^+^ T cells and naive CD4^+^ T cells) were decreased. While these transcriptomic estimates cannot establish direct causality, these findings suggest that the altered immune landscape may be associated with Mtb-related immune evasion. Collectively, these observations support the notion that Mtb infection is not merely a localized pulmonary pathological process but is associated with systemic host transcriptomic remodeling (Khan et al., 2020; Moreira-Teixeira et al., 2020; Cohen et al., 2022).

Based on the transcriptomic data, we identified two core genes, *SP110* and *CCL5*. Incorporating *P2RX7*, a key TB susceptibility gene in Asian populations as reported in the literature, these three were ultimately established as candidate genes. By comparing the Xinjiang IPTB cohort (including both treated and untreated cases) with a control group, this study evaluated the association between the polymorphisms of these candidate genes and the genetic susceptibility to IPTB in the Xinjiang region.

### 5.1 Association between *SP110* gene polymorphisms and IPTB susceptibility

*SP110* is the human homolog of the mouse TB resistance gene *Ipr1*. As a transcriptional regulator, it plays a crucial immunomodulatory role in macrophages (Pan et al., 2005).It downregulates the production of tumor necrosis factor-αby modulating NF-κB activity and simultaneously upregulates anti-apoptotic gene expression, thereby suppressing interferon-γ-mediated mononuclear macrophage death (Nakamura et al., 2024). *SP110* positively regulates macrophage inflammation and interferon responses, providing a novel perspective for elucidating the mechanisms of the TB immune response and granuloma formation (Zhou et al., 2017). Concurrently, the polymorphisms of this gene exhibit significant ethnic heterogeneity regarding TB susceptibility: the rs722555 locus increases susceptibility in Mongolian and Chongqing Han populations but shows no association in southern Chinese populations (Cong et al., 2010; Zhou et al., 2017; Cui et al., 2022); rs9061 is associated with plasma TNF-α levels in individuals with latent infection (Lei et al., 2012); rs11556887 increases the risk of disease onset in Asian populations, whereas large-sample studies suggest that rs1135791 may confer a protective effect (Chang et al., 2018). Conversely, a comprehensive meta-analysis of 10,624 participants revealed no significant association between *SP110* rs9061 and tuberculosis susceptibility in Asian populations, strongly arguing against a major genetic effect at this locus (Zhang et al., 2017). These inconsistencies across studies suggest that the role of *SP110* polymorphisms in TB susceptibility is highly population-specific and dependent on ethnic background, cohort characteristics, and disease phenotype, highlighting the need to validate genetic associations in distinct regional cohorts.

This study investigates the association between the SP110 gene and IPTB in the multiethnic population of Xinjiang. By systematically comparing univariate and multivariate models, we explored the potential confounding effects of baseline demographic characteristics on genetic susceptibility to the disease.

In assessing the risk of IPTB (untreated group vs. control group), the differences observed in the preliminary univariate analysis did not retain statistical significance after FDR correction for multiple testing. Nevertheless, multivariate logistic regression analysis indicated that, after controlling for confounding variables such as age, region, and ethnicity, an increased dose of the A allele at the rs9061 locus yielded a nominally significant, suggestive association with a reduced risk of IPTB. Furthermore, due to the limited subgroup sample sizes, ethnic interaction tests and the Breslow–Day test for homogeneity did not reveal a significant modifying effect of ethnicity on this locus, though this should not be interpreted as absolute proof of homogeneity across ethnic groups. The CMH stratified analysis suggested an exploratory association between this locus and IPTB. Taken together, these adjusted results indicate that the A allele may act as a suggestive factor associated with reduced susceptibility to IPTB in the population of this region. Importantly, these nominally significant multivariable and CMH results do not overcome the multiple-testing issue, and all findings regarding rs9061 must be considered strictly exploratory. They also highlight that, in genetic association studies of complex infectious diseases such as tuberculosis, univariate analyses may be readily confounded by environmental and demographic variables, and that multivariate adjustment is an important approach for exploring potential genetic effects rather than establishing definitive ones.

When evaluating treatment status (untreated group vs. treated group), the findings further highlighted the importance of balancing baseline variables. Although univariate analysis suggested a nominal association between rs9061 and treatment status, multivariate analysis indicated that, after adjusting for potential confounding, the genetic association of rs9061 was markedly attenuated and exhibited only a weak marginal trend. The CMH stratified test also showed no statistical significance. The differences observed initially may thus largely reflect false-positive findings driven by imbalances in non-genetic baseline characteristics. Based on the current study sample, there is insufficient statistical evidence to support a cross-sectional association between the rs9061 polymorphism and subgroups defined by treatment history.

The multiethnic population of the Xinjiang region provides a valuable observational window for gene–environment interactions. This study showed that rs9061 allele frequencies differed between Uyghur and Han participants, reflecting differences in underlying genetic backgrounds. However, neither the multivariate interaction test nor the Breslow–Day test revealed statistically significant interaction effects. Despite objective differences in baseline allele distributions between the two ethnic groups, due to the limited subgroup sample sizes, this absence of statistical significance cannot be interpreted as demonstrating that the biological effect of the rs9061 A allele is completely homogeneous across ethnic strata. This study showed that rs9061 allele frequencies differed between Uyghur and Han participants, reflecting differences in underlying genetic backgrounds.

The rs11556887 and rs1135791 loci did not demonstrate statistically significant differences.

### 5.2 Association between *P2RX7* gene polymorphisms and IPTB susceptibility

The *P2RX7* receptor, encoded by a gene widely expressed in immune cells such as macrophages, serves as an ion channel gated by high concentrations of extracellular ATP. It triggers core immune defense mechanisms, including inflammasome assembly and the release of pro-inflammatory cytokines, by regulating K^+^ efflux alongside Ca^2+^ and Na^+^ influx (Mishra et al., 2021). However, the *P2RX7* gene is highly polymorphic, and SNP mutations within its classical regions significantly alter receptor function. For instance, the classic mutation rs3751143 leads to a loss-of-function of the receptor, thereby significantly increasing an individual’s susceptibility to Mtb (Chaudhary et al., 2018; Chi et al., 2019; Taheri et al., 2019; Keikha and Karbalaei, 2022). In recent years, multiple cross-population studies, including those focused on Asian cohorts, have confirmed that the rs3751143 polymorphism is closely associated with TB incidence (with its C allele acting as a significant risk factor), and it is considered a crucial potential biomarker for predicting the risk of TB onset (Chaudhary et al., 2018; Chi et al., 2019)t. In contrast, the role of gain-of-function mutations, such as rs1718119, in TB immunity is more complex, and current epidemiological evidence remains controversial (Chi et al., 2019; Taheri et al., 2019).

This study investigated the associations of *P2RX7* rs1718119 and rs3751143 with IPTB susceptibility. The results indicated that, in both the overall case-control analysis and the stratified analysis based on clinical characteristics, there were no statistically significant differences in the genotype and allele frequencies of these two loci between the IPTB group and the control group. However, cross-population comparisons revealed significant differences in the genotype and allele distributions of rs1718119 between the Uyghur and Han populations, whereas no significant ethnic differences were observed for rs3751143 between the two groups. Our findings suggest that the *P2RX7* rs1718119 and rs3751143 loci may not directly influence susceptibility to IPTB in specific populations, but they highlight the distinct distribution characteristics of this gene across populations with different genetic backgrounds. When conducting research on host genetics in tuberculosis, it is crucial to fully consider population-specific genetic characteristics, as inter-ethnic genetic variations can significantly impact the generalizability of genetic association findings.

### 5.3 Association between *CCL5* gene polymorphisms and IPTB susceptibility

The *CCL5* gene encodes RANTES, a key chemokine that serves as a core component of immunomodulation and inflammatory responses (Sheng and Qi, 2020). By recruiting and activating T cells and monocytes, RANTES plays a vital role in leukocyte migration during the initial stages of Mtb infection (Méndez-Samperio, 2008). Research indicates that *CCL5* gene variants may influence individual susceptibility to Mtb by modulating T-cell functions (such as the preferential recruitment of Th2 cells) and possess the potential to inhibit intracellular bacterial growth (Sheng and Qi, 2020). Clinically, polymorphisms of this gene are closely associated with the risk of early-onset and active TB (Varzari et al., 2018). Furthermore, fluctuations in plasma RANTES levels following treatment suggest its potential as a biomarker for therapeutic monitoring. Regarding the rs2107538 locus, although it exhibits a significant association with susceptibility in Indian and Caucasian populations, subgroup analyses in East Asian and South African populations have yielded negative results (Mishra et al., 2012; Sheng and Qi, 2020). Such inter-population discrepancies highlight complex gene-gene and gene-environment interactions. Therefore, future studies should employ stratified analyses based on factors such as age, ethnicity, and environmental exposure to further elucidate the underlying genetic pathogenic mechanisms.

This study found no significant association between the *CCL5* rs2107538 polymorphism and pulmonary TB risk. Genotype (G/G, G/A, A/A) and allele distributions were similar between case and control groups, with the A allele showing no protective or risk-enhancing effect. Stratified analysis confirmed these results remained consistent regardless of treatment history. Ultimately, due to the lack of statistical significance in both overall and subgroup populations, the rs2107538 locus appears to hold little clinical value as a molecular biomarker for predicting TB risk within this specific regional population.

## 6 Limitations and future directions

Several limitations of this study should be acknowledged. First, we did not further sub-classify the untreated group (e.g., asymptomatic spontaneously healed vs. symptomatic untreated) or the treated group (e.g., intervention-healed vs. intervention with recurrent symptoms), because current domestic and international clinical guidelines lack explicit definitions and standardized diagnostic boundaries for these specific sub-populations of IPTB; given the retrospective nature of our data, such stratification without guideline-backed criteria would have introduced significant classification bias. Second, while treatment history was ascertained through combined review of medical archives and patient/family interviews, we acknowledge that a degree of recall bias or missing historical documentation is inherent in retrospective reviews. Third, the cross-sectional case-control design demonstrates only an association between gene polymorphisms and IPTB at a single time point and cannot establish causal relationships. Fourth, the bioinformatic analyses were based on retrospective GEO data, and differences between the public database samples and the local Uyghur and Han populations enrolled in this study—in population background, specimen collection procedures, and inclusion/exclusion criteria—limit the generalizability of the database-derived immune cell infiltration profiles and differentially expressed gene results. Fifth, the limited subgroup sample size may have reduced the statistical power of our analyses. Although FDR correction was applied to account for multiple testing, the risk of false-positive findings cannot be fully excluded. Sixth, although we accounted for major ethnic backgrounds, possible residual population stratification cannot be entirely ruled out. Seventh, there is a lack of independent model validation; therefore, our findings should be interpreted with caution until verified in external cohorts. The lack of standardized definitions for these IPTB subgroups highlights an important gap in current clinical practice. In our future prospective studies, we aim to establish clear diagnostic boundaries for “spontaneously healed” versus “treated” IPTB. By combining detailed clinical histories with longitudinal CT imaging, alongside larger multi-center cohorts for external validation, we hope to further elucidate the distinct biological behaviors and clinical outcomes of these specific subgroups, ultimately providing more precise evidence for individualized clinical management.

## 7 Conclusion

Based on the Xinjiang population sample in this study, the A allele at the rs9061 locus of the *SP110* showed a nominal association with decreased susceptibility to IPTB in the univariate analysis, which did not remain significant after FDR correction. Although this association remained nominally significant after multivariate adjustment for potential confounders, these adjusted results do not overcome the multiple-testing issue. Taken together, these findings should be considered exploratory and suggestive of a potentially protective effect for reduced IPTB susceptibility. In contrast, no significant associations were observed between *CCL5* or *P2RX7* polymorphisms and genetic susceptibility to IPTB.

## Data Availability

The datasets presented in this study can be found in online repositories. The names of the repository/repositories and accession number(s) can be found in the article/**Supplementary Material**.
