# Supplementary material for: Association analysis of CCL5, P2RX7, and SP110 gene polymorphisms with inactive pulmonary tuberculosis
Source: Front Cell Infect Microbiol. 2026 Oct 2;16:1937385. doi: 10.3389/fcimb.2026.1937385 (PMC13671932; doi:10.3389/fcimb.2026.1937385)
Supplement: Supplementary file 1 [file Table1.docx]

**1 Laboratory Reagents and Instruments**

**Table1 Laboratory reagents and instruments**

| Reagents/Consumables/Instruments | Manufacturer | Cat.No |
| --- | --- | --- |
| Solution-based DNA Extraction Kit | Junnuode | —— |
| Agarose | BioFroxx | 1110GR500 |
| Super Red Nucleic Acid Dye | Biosharp | BS354B |
| D2000 DNA Marker | Biosharp | BL102A |
| Primers | General Biol | —— |
| 2× Taq plus PCR Master Mix (with dye) | Biosharp | BL553C |
| High-speed Refrigerated Centrifuge | LABGIC | L-CM-1524R |
| Vortex Mixer | Haimen Kylin-Bell Lab Instruments Co., Ltd | GL-88B |
| Dry Bath Incubator | Hangzhou Meisheng Instrument Co., Ltd | Model K20 |
| Nucleic Acid Quantifier | Hangzhou Allsheng | Nano-100 |
| Electrophoresis Power Supply | Beijing Liuyi Instrument Factory | Model DYY-6C |
| Electrophoresis Tank | Beijing Liuyi Instrument Factory | DYCZ-21 |
| Gel Imaging System | LABGIC | L-GIS-A |

**2 Experimental Methods**

**2. 1 Genomic DNA Extraction and Quality Assessment Genomic**

**Genomic DNA Extraction**

**(1) Sample processing and DNA quantification** Prepare a 1× red blood cell (RBC) lysis buffer for the frozen anticoagulated blood samples. Thaw the anticoagulated whole blood to room temperature and mix by inversion. Transfer 200 μL of the blood into a centrifuge tube containing 600 μL of RBC lysis buffer. Invert the tube 6–8 times and gently tap the wall while inverted to ensure thorough mixing. Let the tube stand at room temperature for 10 min, inverting to mix several times during this incubation. Centrifuge at 12,000 rpm for 1 min at room temperature. Discard the red supernatant and invert the tube on absorbent paper to remove any residual blood from the rim. Add an additional 300 μL of RBC lysis buffer, invert several times, let stand for 2 min, and centrifuge again at 12,000 rpm for 1 min at room temperature before discarding the red supernatant.

Next, add 300 μL of nuclear lysis buffer (pre-warmed to 50°C). Gently pipette the mixture up and down 10–15 times to disperse the cell pellet. Avoid vigorous pipetting to prevent shearing the genomic DNA, and ensure no air bubbles are introduced. Incubate the mixture in an incubator at 60–65°C for 45 min until the solution becomes clear. Add 150 μL of protein precipitation solution and vortex for 25 s. Centrifuge at 12,000 rpm for 5 min at room temperature.

Carefully transfer the supernatant (approximately 450 μL) to a new centrifuge tube. Add an equal volume of isopropanol (450 μL) and mix gently by inverting 30 times, or until a white, flocculent DNA precipitate appears. Centrifuge at 12,000 rpm for 5 min at room temperature; a white DNA pellet will be visible at the bottom of the tube. Discard the supernatant. Add 500 μL of 70% ethanol and invert several times to wash the DNA. Centrifuge at 12,000 rpm for 3 min at room temperature and carefully discard the supernatant (if the DNA pellet is loosely attached to the wall, take caution not to pour it out). Repeat this 70% ethanol washing step once.

Centrifuge briefly at 12,000 rpm for 30 s at room temperature. Carefully pipette out the residual solution, leave the tube cap open, and let it air-dry at room temperature for 1 min. Finally, add 50 μL of ddH2O to dissolve the DNA pellet. The DNA concentration and purity of the solution were measured using a nucleic acid and protein quantifier.

**(2) Quality assessment of extracted DNA via electrophoresis** Agarose gel electrophoresis was performed to assess DNA integrity. A 1% agarose gel was prepared by dissolving agarose in 1× TAE buffer, followed by the addition of a nucleic acid dye. The extracted DNA sample was mixed with 1 μL of 6× loading buffer and loaded into the gel wells. Simultaneously, 5 μL of a DNA marker was loaded into an adjacent well. Electrophoresis was conducted in 1× TAE buffer at a constant voltage of 120 V for 30 min. Upon completion, the gel was observed, and the results were captured and saved using a gel imaging system. The quality-checked DNA samples were stored at -20°C for subsequent use.

**2. 2 Primer Design and Synthesis**

Based on the template sequences encompassing the target SNPs, the primer sequences listed in the subsequent table were designed using Primer Premier 5.0 software. The primers were synthesized utilizing the solid-phase phosphoramidite triester method. In this approach, DNA is initially immobilized on a solid-phase support and subsequently subjected to a four-step cyclic reaction comprising deprotection, coupling, capping, and oxidation. Through this continuous cycle, nucleotide monomers are sequentially linked one by one until the full-length sequence synthesis is successfully achieved.

**Table2 Primer sequences and expected product sizes for PCR**

| Primer Name | Sequence (5' to 3') | Amplicon Size (bp) | Annealing Temperature (°C) |
| --- | --- | --- | --- |
| rs9061-F | CTTGAGCTAAGCGGTAT | 568 | 57.6 |
| rs9061-R | GTCAGTGAGCCTGGAAC |  |  |
| rs11556887-F | GGCGACTCACTCAGGAT | 370 | 57.6 |
| rs11556887-R | GGGCCACAAATGGATAA |  |  |
| rs1135791-F | GCAGGCATCTGTCTTCA | 627 | 60.7 |
| rs1135791-R | TCAGTCAGGCTTTAGGG |  |  |
| rs2107538-F | GGAGTGGCAGTTAGGACA | 496 | 57.6 |
| rs2107538-R | AGTGGCTGGCACAGATAA |  |  |
| rs1718119-F | ACCCTCACCTTGTATGGA | 580 | 57.6 |
| rs1718119-R | ATGGACTCGCACTTCTTC |  |  |
| rs3751143-F | CCTGAATGAGGCAATG | 483 | 57.6 |
| rs3751143-R | AAGGGCTCCTGGTAGA |  |  |

**2. 3 Polymerase Chain Reaction (PCR)**

The PCR reaction mixture was prepared according to Table 4, and the PCR tubes were placed into a thermal cycler to execute the amplification protocol outlined in Table 5.

The amplified products were subsequently verified via gel electrophoresis. Briefly, a 1% agarose gel was prepared by dissolving agarose in 1× TAE buffer supplemented with a nucleic acid dye. A 5 μL aliquot of the PCR product was mixed with 1 μL of 6× loading buffer and loaded into the gel wells, alongside 5 μL of a DNA marker. Electrophoresis was conducted in 1× TAE buffer at a constant voltage of 120 V for 30 min. Upon completion, the gel was visualized, and the images were recorded using a gel imaging system.

**Table 3. Components and volumes of the PCR amplification reaction mixture**

| Component | Volume (μL) |
| --- | --- |
| DNA | 2 |
| Forward Primer（10 μM） | 1 |
| Reverse Primer（10 μM） | 1 |
| 2×Taq plus PCR Master Mix | 25 |
| ddH_2_O | 21 |

**Table4 Optimized thermal cycling parameters for PCR amplification**

| Step | Temperature | Time | Cycles |
| --- | --- | --- | --- |
| Initial denaturation | 94℃ | 3 min | 1 |
| Denaturation | 94℃ | 30 s | 35 |
| Annealing | Refer to primer table | 30 s |  |
| Extension | 72℃ | 38 s |  |
| Final extension | 72℃ | 5 min | 1 |

Note: After amplification, the PCR products were maintained at 4°C.

**2.4 Gene Sequencing and Genotyping Analysis**

The aforementioned positive PCR products were subjected to Sanger sequencing. Genotyping results were determined by reviewing the sequencing electropherograms obtained after pre-sequencing sample purification, sequencing reactions, purification of the sequencing products, and loading onto the sequencer to acquire the base sequences.
