# Supplementary material for: Association analysis of CCL5, P2RX7, and SP110 gene polymorphisms with inactive pulmonary tuberculosis
Source: Front Cell Infect Microbiol. 2026 Oct 2;16:1937385. doi: 10.3389/fcimb.2026.1937385 (PMC13671932; doi:10.3389/fcimb.2026.1937385)
Supplement: Supplementary file 2 [file Table2.docx]

**1. GEO Preprocessing and Data Integration** This study integrated five microarray datasets (GSE19435, GSE19439, GSE19443, GSE28623, and GSE29536). After extracting the common genes across multiple datasets, a log2 transformation was performed on the raw data that were on a linear scale. Subsequently, quantile normalization was applied to standardize the merged expression matrix. To evaluate batch effects visually, the removeBatchEffect function from the *limma* package was used to correct the data specifically for downstream Principal Component Analysis (PCA) visualization.

**2. Batch Effect Processing**

- **For Visualization:** Direct batch effect correction was performed on the normalized data using the removeBatchEffect function in the *limma* package. However, these explicitly batch-corrected data were used exclusively for visualization purposes (e.g., PCA).
- **For Differential Expression Analysis:** Differential analysis was conducted directly on the normalized expression matrix using the R package *limma*. Instead of using the pre-corrected data, the dataset batch information was incorporated as a covariate into the linear model to statistically control for batch effects. The statistical thresholds for screening differentially expressed genes (DEGs) were set at an Adjusted P-value < 0.05 and |log2(Fold Change)| > 1.

**3. Machine Learning Parameters**

- **LASSO Model:** The LASSO regression model was constructed using the *glmnet* package (family = "binomial"). A 10-fold cross-validation (nfolds = 10) was executed via the cv.glmnet function to dynamically evaluate the regularization path. The penalty coefficient yielding the minimum error (lambda.min) was selected as the optimal hyperparameter, and features with non-zero coefficients were extracted as candidate biomarkers.
- **Random Forest Model:** The Random Forest model was developed utilizing both the *caret* and *randomForest* packages, with a random seed set to ensure reproducibility. A 10-fold cross-validation was configured via trainControl for hyperparameter optimization. The total number of decision trees was fixed at 500 (ntree = 500), and a grid search was employed to set the number of variables randomly sampled as candidates at each split (mtry) to the square root of the total number of features. Finally, gene importance was evaluated based on the MeanDecreaseGini metric, and the top 10 genes were extracted as core biomarkers.
- **SVM-RFE Model:** The Support Vector Machine-Recursive Feature Elimination (SVM-RFE) model was constructed based on a 5-fold cross-validation. Computational parameters were set to halve.above = 100 and k = 5 to perform dynamic recursive elimination (i.e., halving the number of eliminated features in each iteration when the remaining feature count exceeded 100). By systematically traversing the feature subsets, the cross-validation error rate was minimized to 0.0612 when 86 features were retained. Based on this minimum error rate, the optimal core gene set was determined and extracted.
