# Supplementary material for: Association analysis of CCL5, P2RX7, and SP110 gene polymorphisms with inactive pulmonary tuberculosis
Source: Front Cell Infect Microbiol. 2026 Oct 2;16:1937385. doi: 10.3389/fcimb.2026.1937385 (PMC13671932; doi:10.3389/fcimb.2026.1937385)
Supplement: Supplementary file 3 [file Table3.docx]

1. Definition of Inactive Pulmonary Tuberculosis

Inactive pulmonary tuberculosis refers to relatively stable tuberculosis-related thoracic imaging changes, with or without a history of tuberculosis, no clinical manifestations associated with pulmonary tuberculosis, evidence of *Mycobacterium tuberculosis* (MTB) infection, negative etiological test results for tuberculosis, and exclusion of pulmonary diseases caused by other reasons.

2. Diagnostic Criteria for Inactive Pulmonary Tuberculosis

(1) History of tuberculosis: Any one of the following three groups of people.

1. Individuals without a clear history of tuberculosis, who are found to have relatively stable tuberculosis-related imaging changes on thoracic imaging examination.
2. Individuals with previously identified relatively stable tuberculosis-related thoracic imaging changes on prior thoracic imaging, who received irregular anti-tuberculosis treatment or no treatment.
3. Patients with a confirmed diagnosis and treatment history of pulmonary tuberculosis, who completed the full course of regular anti-tuberculosis treatment and were assessed as cured or treatment completed at treatment outcome evaluation.

(2) Clinical manifestations: No suspected clinical manifestations of pulmonary tuberculosis such as cough, expectoration, fever, night sweats, chest pain, or weight loss.

(3) Tuberculosis etiological examination: Microscopic examination of 3 sputum smears with acid-fast staining, mycobacterial culture, and nucleic acid tests are all negative.

(4) MTB infection test meeting one of the diagnostic criteria for latent tuberculosis infection (LTBI).

1. In the absence of interference from BCG vaccination or non-tuberculous mycobacteria (NTM), an average diameter of induration ≥5 mm on the purified protein derivative (PPD) skin test indicates established MTB infection.
2. In BCG-vaccinated areas or areas with NTM infection, an average induration diameter ≥10 mm on the PPD skin test is the criterion for MTB infection.
3. For HIV-positive individuals or those receiving immunosuppressant therapy for more than 1 month, as well as children ≤5 years old without BCG vaccination who have had close contact with patients with active pulmonary tuberculosis, an average induration diameter ≥5 mm on the PPD skin test indicates MTB infection.
4. Positive recombinant *Mycobacterium tuberculosis* fusion protein (EC) skin test, also known as the novel tuberculin skin test (creation tuberculin skin test, C-TST), indicates MTB infection.
5. A positive interferon-γ release assays (IGRA) test is regarded as evidence of MTB infection.

(5) Thoracic imaging examination: Presence of one or more imaging features of inactive pulmonary tuberculosis.

1. Imaging features of pulmonary lesions: (1) Calcified lesions: Tuberculous lesions are partially or completely calcified, with lesion density close to that of the ribs and sharp, well-defined margins. (2) Fibrous lesions: Present as localized stellate, fine linear or thick linear opacities with clear margins. (3) Sclerosing nodules: Nodular, irregular in shape, high density with clear margins, or accompanied by calcification. (4) Cleaned cavity: Thin-walled cavity with wall thickness less than 3 mm, smooth inner wall, no intracavitary content, surrounded by fibrous lesions of varying amounts. (5) Pulmonary sclerosis: Lesions manifest as segmental or lobar soft-tissue density opacities with relatively well-defined borders, higher density than muscle tissue, with or without calcification; dilated bronchi or fibrous cavities can be seen, without focal liquefactive necrosis. Mediastinal, tracheal and hilar displacement is visible in most cases, and fibrous and sclerotic changes can be seen in other lung regions.
2. Imaging features of pleural lesions: Localized or extensive pleural thickening and adhesion, which may be accompanied by calcification of various morphologies.
3. Imaging features of lymph node lesions: Complete or partial calcification of hilar and mediastinal lymph nodes.
4. Imaging features of bronchial lesions: Narrowed bronchial lumen with smooth inner wall, or accompanied by mucosal calcification; intrapulmonary lesions show complete absorption, fibrosis or sclerotic changes.

Diagnosis of inactive pulmonary tuberculosis: All items (1) to (4) of the diagnostic criteria for inactive pulmonary tuberculosis AND any one feature in item (5) must be satisfied, with other pulmonary diseases excluded.
